# Enhancing Maize Productivity and Soil Health under Salt Stress through Physiological Adaptation and Metabolic Regulation Using Indigenous Biostimulants

**DOI:** 10.3390/plants12213703

**Published:** 2023-10-27

**Authors:** Redouane Ouhaddou, Abdelilah Meddich, Chayma Ikan, Rachid Lahlali, Essaid Ait Barka, Mohammad-Reza Hajirezaei, Robin Duponnois, Marouane Baslam

**Affiliations:** 1 Center of Agrobiotechnology and Bioengineering, Research Unit Labelled CNRST (Centre AgroBiotech-URL-7 CNRST-05), Cadi Ayyad University, Marrakesh 40000, Morocco; 2 Plant Physiology and Biotechnology Team, Laboratory of Agro-Food, Biotechnologies and Valorization of Plant Bioresources (AGROBIOVAL), Department of Biology, Faculty of Science Semlalia, Cadi Ayyad University (UCA), Marrakesh 40000, Morocco; 3 Department of Plant Protection, Phytopathology Unit, Ecole Nationale d’Agriculture de Meknès, Km10, Rte Haj Kaddour, BP S/40, Meknès 50001, Morocco; 4 Plant Pathology Laboratory, AgroBioSciences, Mohammed VI Polytechnic University, Lot 660, Hay Moulay Rachid, Ben Guerir 43150, Morocco; 5 Unité de Recherche Résistance Induite et Bio-Protection des Plantes-EA 4707, Université de Reims Champagne-Ardenne, 51100 Reims, France; 6 Leibniz Institute of Plant Genetics and Crop Plant Research (IPK), Molecular Plant Nutrition, Department of Physiology and Cell Biology, OT Gatersleben, Corrensstrasse 3, D-06466 Seeland, Germany; 7 Laboratoire des Symbioses Tropicales & Méditerranéennes UMR 113 IRD/CIRAD/INRAe/SupAgro Montpellier/UM Campus International de Baillarguet TA A-82/J, CEDEX 5, 34398 Montpellier, France; 8 GrowSmart, Seoul 07516, Republic of Korea

**Keywords:** native biostimulants, physiological adaptation, metabolic regulation, arbuscular mycorrhizal fungi, *Bacillus* sp., compost, salinity, soil structure

## Abstract

Salinity poses a persistent threat to agricultural land, continuously jeopardizing global food security. This study aimed to enhance sweet corn (SC) fitness under varying levels of salinity using indigenous biostimulants (BioS) and to assess their impacts on plant performance and soil quality. The experiment included control (0 mM NaCl), moderate stress (MS; 50 mM NaCl), and severe stress (SS; 100 mM NaCl) conditions. Indigenous biostimulants, including compost (C), *Bacillus* sp., *Bacillus subtilis* (R), and a consortium of arbuscular mycorrhizal fungi (A) were applied either individually or in combination. Growth traits, physiological and biochemical parameters in maize plants, and the physico–chemical properties of their associated soils were assessed. SS negatively affected plant growth and soil quality. The RC combination significantly improved plant growth under SS, increasing aerial (238%) and root (220%) dry weights compared to controls. This treatment reduced hydrogen peroxide by 54% and increased peroxidase activity by 46% compared to controls. The indigenous biostimulants, particularly C and R, enhanced soil structure and mineral composition (K and Mg). Soil organic carbon and available phosphorus increased notably in C-treated soils. Furthermore, RC (437%) and CAR (354%) treatments exhibited a significant increase in glomalin content under SS. Indigenous biostimulants offer a promising strategy to mitigate salinity-related threats to agricultural land. They improve plant fitness, fine-tune metabolism, and reduce oxidative stress. In addition, the biostimulants improved the soil structure and mineral composition, highlighting their potential for reconstitution and sustainability in salt-affected areas. This approach holds promise for addressing salinity-related threats to global food security.

## 1. Introduction

Soil is an essential resource for sustaining the world’s growing population, which is projected to surpass 9.8 billion by 2050 [1]. Among the most threatening abiotic factors negatively affecting agricultural systems is salinity stress [2]. Currently, salinity stress affects approximately 160 million hectares (Mha) of cultivated land worldwide, resulting in a productivity loss of 1.5 Mha annually [3]. Soil salinity affects one billion hectares in over 100 countries [4], making it a widespread problem. Salinity significantly impacts plant survival, growth, and productivity by restricting nutrient availability through soil complexation, inducing physiological changes, and impairing antioxidant function [5,6]. Additionally, salinity also influences protein development, lipid digestion, and photosynthesis [7]. The main detrimental effects of soil salinity are nutrient deficiency, reduced osmotic pressure, and decreased soil water uptake [8,9]. The presence of excess Na^+^ and Cl^−^ ions leads to oxidative and osmotic pressure, disruption of cellular homeostasis, and uncoupling of key biochemical and physiological processes [10,11,12]. Plants adapted to grow in saline conditions can reduce the damage caused by Na^+^ by limiting its uptake, shifting Na^+^ from shoots to roots, and expelling Na^+^ loads from their root cells [13].

Rhizospheric microorganisms have shown promise as biological tools to enhance the growth of different crops under abiotic constraints [14,15,16]. Microorganisms such as arbuscular mycorrhizal fungi (AMF) and plant growth-promoting bacteria (PGPR) have been shown to enhance plant growth and development, particularly in saline soils [4,17]. The symbiotic relationship between AMF and plant roots improves plant growth and vigor and protects host plants from damage caused by soil salinization [18,19]. AMF secrete substances such as glomalin, which bind soil particles and stabilize aggregates, thereby impacting soil structure [20]. PGPR reduce the negative effects of salinity by forming biofilms [21], accumulating osmolytes in the plant cell cytoplasm, and maintaining cell turgor [22]. In addition, compost offers a sustainable approach to improving soil quality. Compost is known for its high porosity, aeration, drainage, and water-holding capacity [23]. Moreover, compost is a valuable source of water-soluble nutrients and serves as an excellent organic fertilizer and soil conditioner [23]. Compost is also a valuable source of water-soluble nutrients that promote plant growth and development in saline soils [7,24].

Sweet corn (*Zea mays* L. var. Saccharata) is widely consumed as a vegetable and fresh food for humans due to its delicious taste and high vitamin content [25,26]. However, unfavorable environmental conditions, particularly salinity stress, limit its spread as a crop [27,28]. Sweet corn is distinguished from other maize varieties by a mutation at chromosome 4 (Su (sugary) locus) [29], which leads to increased synthesis of soluble sugars and polysaccharides in the seed endosperm. The objective of this study is to evaluate the efficacy of microorganisms isolated from a (semi)arid region, with or without local compost, in improving sweet corn tolerance to different salinity levels. The main parameters used to assess the behavior of the BioS applied to sweet corn plants under saline conditions include growth traits, physiology, and soil quality.

## 2. Results

### 2.1. Effects of Salt Stress and BioS on Growth and Mycorrhization of Maize Plants

**Figure 1:**
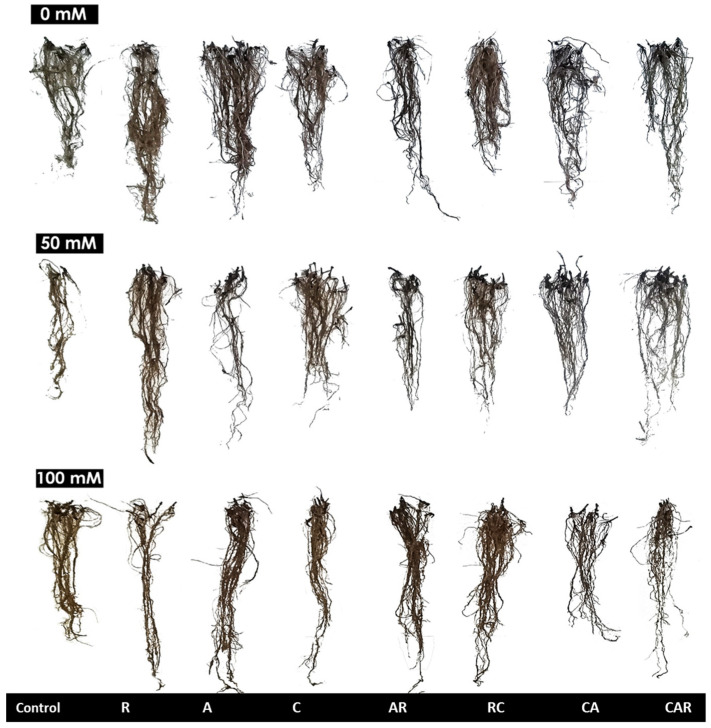
Effect of salinity (0, 50, and 100 mM NaCl) on the root system of sweet corn. The treatments included control plants, sweet corn plants amended and inoculated with single biostimulants (compost (C), native arbuscular mycorrhizal fungi consortium (A), and bacterial consortium (R)), double combinations (AR: AMF + R consortia, RC: R consortium + compost, CA: compost + AMF consortium), and a triple combination (CAR: compost + AMF + R consortia).

The growth parameters of the maize plants, including shoot (SDW) and root (RDW) dry weight, plant height (SH), root length (RL), and leaf number (LN), were significantly (*p* < 0.01) affected by salt stress and the BioS treatments (Figure 1, Table 1 and Table S1).

The untreated sweet corn plants showed negative effects under salt stress, but the addition of compost alone or combined with the bacterial consortium and double application of R and mycorrhizal consortia, significantly improved SDW under 100 mM NaCl compared to the stressed control plants (*p* < 0.05). The interaction between CxRxSS had a significant effect (*p* < 0.05) on the SDW (Table S1). Similarly, the RDW of the plants treated with the RC combination was significantly higher than that of the untreated plants. The SH was significantly improved by the C and A combination under SS conditions compared to the stressed control. The plants inoculated with R also showed a significant increase in root elongation (RL) (*p* < 0.05) under 50 mM NaCl compared to the control (Figure 1). Under MS conditions, the highest number of leaves (LN) was observed in the plant treated with compost and the bacterial consortium in the soil (Table 1). Similar results were observed under SS conditions compared to the stressed control. Furthermore, aerial elongation was significantly increased (*p* < 0.05) in the plants inoculated with CA under SS conditions compared to the control plants.

The mycorrhization parameters were affected by salt stress, as shown in Table 1. Non-mycorrhized plants showed no mycorrhizal colonization upon microscopic examination, whereas the mycorrhization frequency was approximately 93% and 86% in the roots of the CA- and CAR-treated plants, respectively, under SS conditions. The intensity of mycorrhization increased in the presence of C (24%) or R (23%) under SS conditions. Salt stress also affected mycorrhizal dependence (MD), which increased significantly in the plants treated with AR (68%) under SS conditions, while those treated with CA (74%) and CAR (75%) exhibited higher values under MS conditions compared to the control plants (Table 1). The results of the MANOVA revealed that the interaction between AxSS had a significant effect (*p* < 0.01) on MF, MI, and MD (Table S1).

### 2.2. Effect of Salt Stress and BioS on Physiological Parameters

The physiological parameters of the plants were affected by salt stress (Table 2). The application of BioS had a positive impact on these parameters under SS conditions. Chlorophyll fluorescence (F_v_/F_m_) was positively influenced by the application of C and R under SS conditions compared to the stressed and untreated plants, although the difference was not significant. Stomatal conductance (g_s_) was significantly improved by the combination of C and R (CR) under MS and SS conditions compared to the respective controls (*p* < 0.05, Table 2). Water content was also significantly improved by C and A compared to the control plants under salt stress conditions (*p* < 0.05, Table 2). The interactions between SSxCxAxR had a significant effect (*p* < 0.01) on F_v_/F_m_ (Table S1).

### 2.3. Effects of Salt Stress and BioS on Corn Biochemistry

**Figure 2:**
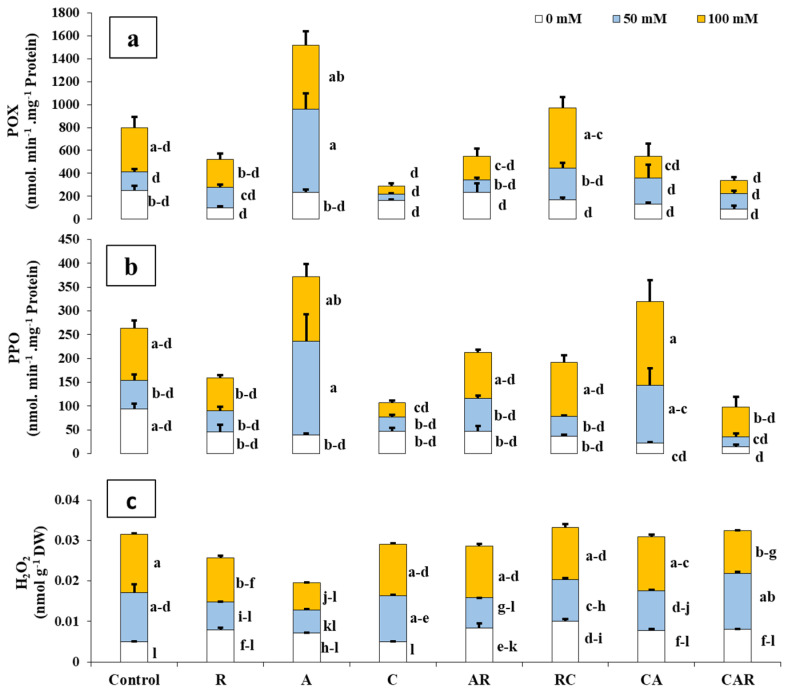
Influence of different salinity levels (0, 50, and 100 mM NaCl) and biostimulant applications on (**a**) POX: peroxidase, (**b**) PPO: polyphenoloxidase, and (**c**) H_2_O_2_: hydrogen peroxide in sweet corn. The treatments included control plants, sweet corn plants amended and inoculated with single biostimulants (compost (C), native arbuscular mycorrhizal fungi consortium (A), and bacterial consortium (R)), double combinations (AR: AMF + R consortia, RC: R consortium + compost, CA: compost + AMF consortium), and a triple combination (CAR: compost + AMF + R consortia). The data represent the mean ± SE of 5 biological replicates. Means with the same letter are not significantly different at *p* < 0.05 (Tukey’s HSD).

Salt stress resulted in alterations in cellular metabolism, specifically affecting POX and PPO activity (Figure 2). Application of A significantly increased POX and PPO activity under MS conditions compared to the stressed control plants (*p* < 0.05, Figure 2a,b). The interaction between SSxA had a significant effect (*p* < 0.001) on POX (Table S1). The accumulation of stress markers, such as H_2_O_2_, increased significantly under salinity conditions (Figure 2c). However, the application of BioS led to a significant decrease in H_2_O_2_ levels, especially in the plants treated with A, CAR, and R by 54%, 26%, and 25%, respectively, compared to their control plants under SS conditions. Similarly, A, R, and AR reduced the negative effect of H_2_O_2_ by 52%, 43%, and 38%, respectively, compared to their control plants under MS conditions.

### 2.4. Effect of Salt Stress and BioS on Soil Physicochemical Properties

The physicochemical properties of the soil were significantly improved after 4 months of maize cultivation compared to the initial state, with BioS having a positive impact at various levels (TOC, TOM, AP, EC, and pH) (*p* < 0.05, Table 3). The combination of C and A or AR significantly improved TOC and TOM under SS conditions. Plants grown in soil amended with CAR, CM, and RC exhibited a significant improvement in AP contents, regardless of the salinity levels. The interactions between SS*R*C had a significant impact (*p* < 0.01) on AP (Table S1). RC and AR treatment also reduced both pH (2%) and EC (35%) under SS conditions compared to the control soil. Salt stress and BioS significantly affected the glomalin content in the soil (Table 3). Under saline conditions and in the presence of the BioS, glomalin increased significantly under both 50 and 100 mM NaCl compared to the control conditions. Under MS, inoculation and/or amendment of SC plants with AR, CA, and CAR resulted in high glomalin levels compared to stressed control plants. Under SS, these values were doubled in the single C, dual (RC and CA), and triple (CAR) combinations compared to the control conditions.

The EDX results revealed that the mineral composition of the soil was affected by the application of BioS and the salinity level (Table 4). Potassium showed a highly significant improvement, especially with R inoculation (151%) and its association with C (78%) (*p* < 0.05) under SS conditions compared to the control soil. In addition, under the same conditions, the soil treated with C showed a remarkable increase in calcium content (34%), whereas the soil treated with RC revealed a reduction of 55% compared to the control. Under SS conditions, CAR- and R-treated soils exhibited a decrease (33%) and an increase (33%) in Mg content, respectively. The AR treatment showed a 70% increase in Fe content compared to the control soil. The interaction between salinity and the triple combination CAR had a significant effect (*p* < 0.001) on K, Ca, Mg, and Fe content (Table S1). R alone or combined with C displayed a significant reduction in Na (about 92%), while AR showed an increase of 19% compared to the control under SS conditions.

**Figure 3:**
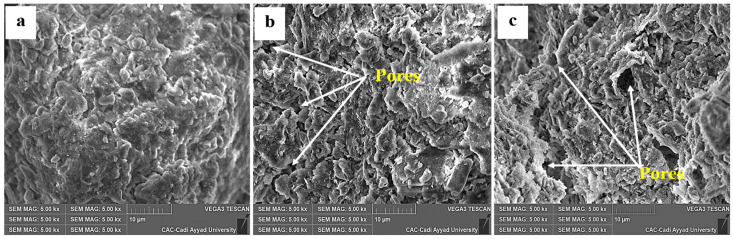
Scanning electron microscopy (SEM) observations of the soil structure of (**a**) control, (**b**) soil treated with compost (C), and (**c**) soil treated with plant growth-promoting bacteria (R).

The SEM results showed that the structure of the sweet corn soil was influenced by the addition of BioS (Figure 3). While the untreated soil showed a normal structure (Figure 3a), the application of BioS led to a noticeable change in the structure based on the pore appearance compared to the control soil (Figure 3b,c).

### 2.5. Principal Component Analysis

**Figure 4:**
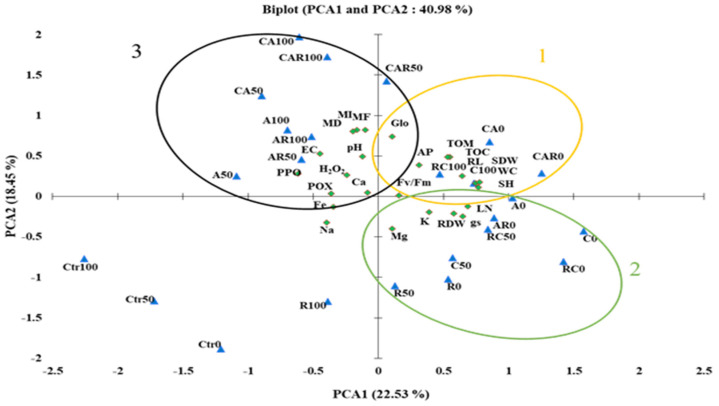
(A) Principal component analysis (PCA) of soil and plant data of sweet corn under control (0 mM NaCl) or stressed (moderate stress; 50 mM NaCl and severe stress; 100 mM NaCl) conditions, subjected to different treatments, Ct: untreated control; R: +PGPR; A: +AMF; C: +compost, and their combination. LN: leaf number; SH: shoot height; RL: root length; RDW: root dry weight; SDW: shoot dry weight; F_v_/F_m_: chlorophyll fluorescence; g_s_: stomatal conductance; WC: water content; MI: mycorrhizal intensity; MF: mycorrhizal frequency; MD: mycorrhizal dependency; H_2_O_2_: hydrogen peroxide; POX: peroxidase activity; PPO: polyphenoloxidase activity; pH: hydrogen potential; EC: electrical conductivity; TOM: total organic matter; TOC: total organic carbon; PA: phosphorus in soil; Glo: Glomalin; Ca: calcium; K: potassium; Na: sodium; Mg: magnesium; Fe: iron.

The study employed PCA analyses to examine the salt stress response and its correlations with the traits analyzed, and to identify the best treatments (Figure 4). The PCA revealed that the traits and treatments were associated (41%) with PCA1 and PCA2, of which PCA1 was the major component (23%). Strong positive correlations were found between growth (SDW, RL, and SH), physiological (F_v_/F_m_ and WC), and soil physico–chemical (AP, Glo, TOM, and TOC) parameters in the presence of CA, CAR, and A under normal conditions (circle 1; yellow color). K and Mg showed a positive correlation with the growth (RDW and LN) and physiological (g_s_) parameters (circle 2; green color) in the presence of AR and RC under normal and MS conditions, respectively. Conversely, the 100 mM NaCl, H_2_O_2_, POX, PPO, and mycorrhization parameters (MD, MI, and MF) were positively correlated with the PCA2 axis (circle 3; black color). Fe and Na were negatively correlated with LN, RDW, K, and Mg on the PCA axis.

## 3. Discussion

The semi-arid to arid Mediterranean regions are facing an aggravated situation due to the impact of climate change, leading to soil quality deterioration that threatens the agricultural sector [30,31,32]. FAOSTAT [3] emphasizes the need for sustainable soil management practices to reverse soil degradation and inform decision makers about climate change adaptation and appropriate irrigation schemes. Sustainable organic agriculture is considered an ecological solution to restore degraded soils to ensure agricultural production and food security [33,34]. Salt stress has been reported to negatively impact the growth and development of several plant species, including sweet corn [29,35,36]. In this study, the negative effects of salt stress on sweet corn fitness were also observed, including a reduction in above and below ground biomass and root elongation. The inhibition of primary root growth under excess salt in the rhizosphere of plants is due to inhibition of cell division and elongation in root epidermal cells [37]. Salt stress disrupts the homeostatic transport of Na^+^ across intracellular and intercellular boundaries, facilitated by NaCl, contributing to the negative effects [38].

Recent interest has focused on harnessing the potential of the rhizosphere microbial community and organic amendments to improve poor soil quality. However, the effectiveness of these approaches is influenced by abiotic stresses (salinity and drought) and soil nutrient depletion conditions, which can affect the ability of AMF and PGPR to colonize and promote plant growth [39]. The results of this study demonstrate a net improvement in growth when treated with compost alone or in combination with R or M. Compost positively affects growth due to its mineral elements, humic substances, and soil structure improvements, with better water and oxygen circulation [40,41]. AMF play a crucial role in nutrient cycling, maintaining soil health and plant growth by enhancing water and nutrient absorption, particularly P, and improving root structure through the development of a large mycelial network in the soil and its association with P-solubilizing microorganisms (PSM) [42].

The results showed the beneficial effects of PGPR, AMF, and/or compost on root system elongation and biomass, which are rarely investigated. This well-developed root architecture is attributed to the stimulation of cell divisions through the synthesis of phytohormones, especially auxin, and exopolysaccharides production, which facilitate access to nutrients in the rhizosphere [43,44] The positive effects on the morphological parameters of the SC plants under salt stress conditions may be related to the high frequency and degree of mycorrhizal roots. These results are consistent with those of Ouhaddou et al. [6]. The mycorrhization rate increased significantly when combined with compost, possibly facilitated by the physiochemical properties of the compost, promoting successful root infection [45,46]. The presence of organic additives (i.e., humic acids) has been shown to promote AMF colonization of the root cortex [47,48]. On the contrary, some authors have reported a blockage of AMF activity when plant requirements are already fulfilled by compost in sufficient quantities [49,50]. In general, it is postulated that the infection rate of AMF increases in soils with limited P availability [51]. Ouhaddou et al. [6] confirmed that intense mycorrhization occurred in lettuce plants treated with compost containing 88 mg/kg of soil P content. Mycorrhizal dependency (MD) was 68% in the MR combination under salt stress conditions. MD is often associated with P and Zn levels [52]. However, the response to AM symbiosis varies from positive to negative depending on the plant genotype, fungus, environmental conditions, and phenological stage of the plant [53].

The symbiosis established with compost and/or PGPRs benefits the growth of sweet corn plants by improving their physiology, photosynthetic activity, and water status. The applied BioS, especially R alone or in combination with compost, positively influenced chlorophyll fluorescence and stomatal conductance under salt stress conditions. This indicated improved photosynthetic activity [54] and efficient CO_2_ uptake leading to the synthesis of organic matter, including proteins and sugars [55]. The increased gas exchange in BioS-treated plants is associated with enhanced absorption and water translocation [56], leading to the regulation of stomatal dynamics (opening and closing) [57,58]. Previous studies have linked improved chlorophyll synthesis to increased uptake of mineral elements, particularly Mg and N [59,60,61]. Chen et al. [62] demonstrated that inoculated plants under 100 mM NaCl upregulated the expression of chloroplast genes (*Rppsb*A and *Rppsb*D), resulting in higher PSII efficiency and increased photosynthetic capacity under salt stress conditions.

The water status of both treated and untreated plants was affected by exposure to high salinity. Mycorrhizal plants (A) or those amended with compost (C) showed a positive response in water content. AMF–plant symbiosis improves water status, facilitates plant growth and photosynthesis, and has a positive effect on relative water content [63,64]. The extensive hyphal extensions of mycorrhizae contribute to higher hydraulic conductivity, even under a low water potential [65,66]. Additionally, higher stomatal conductance and transpiration improve water status [64]. Studies have indicated that aquaporins, proteins involved in improving water status, play a crucial role in mycorrhized plants under extreme conditions [67,68].

Salt stress not only negatively impacts the growth parameters of plants but also disrupts their biochemical metabolism. This disruption results in an increased production of ROS and a high rate of lipid peroxidation [69]. Our study reveals that maize exposed to 100 mM NaCl exhibits a significant increase in H_2_O_2_ levels in their leaves, indicating oxidative damage. However, selected BioS have a beneficial effect in reducing this stress marker by activating the respective enzyme systems. Among the treatments, sweet corn plants inoculated with A demonstrated the most significant reduction in H_2_O_2_ levels. It has been reported that AMF inoculation increases the activities of antioxidant enzymes in salt-stressed maize plants, leading to faster scavenging of ROS and prevention of oxidative stress [56]. Our results show that salt stress, as well as individual A inoculation, further increases the antioxidant activity of POX and PPO enzymes. These findings align with those obtained by Khan et al. [2] and may be attributed to the overexpression of key genes following colonization of the root cortex [70]. It is essential to note that any positive or negative effects of salinity or BioS on the growth and development of sweet corn plants are closely related to the physicochemical and biological properties of the soil.

Previous studies have highlighted that high concentrations of salts in the soil can reduce the availability of water and essential mineral elements for plants and microorganisms [71,72,73]. Soluble salts in soil often negatively influence the organic matter content and soil stability [74]. Due to their immobilization, saline soils contain limited organic matter and mineral nutrients that are challenging for plant roots to access [33]. Recent research has shown that enriching saline soil with BioS can lead to an improvement in soil quality and structure [7]. The combination of C and/or A and/or R in our study positively affected the TOM and TOC levels, as well as the mineral status of the soil. The increase in TOM and TOC can be attributed to the relative richness of the compost in organic matter and the ability of the selected strains to metabolize various root exudates (i.e., amino acids, carbohydrates, and organic acids) secreted by the plant [75]. These findings are consistent with our recent publication on lettuce plants under salt stress [6]. Other studies have also demonstrated the beneficial effects of local composts on crop growth and soil fertility [76]. Compost can enhance soil structural stability, promote aggregate formation, and improve hydraulic conductivity [77,78]. In our present work, we observed a well-aerated soil structure, indicated by the presence of pores, in the soil treated with compost or in combination with microbes compared to the untreated soil. This observation was supported by the scanning electron microscopy results obtained by Archanjo et al. [79], which showed the formation of pores at the interface between the C matrix and organo–mineral aggregates. The modification of soil structure observed in our study after the addition of compost and/or microorganisms might be attributed to the secretion of exopolysaccharides (EPS) and siderophores by PGPRs, which are essential for preserving soil health [16,80]. Glomalin, as found in our study, contributes to aggregate stability and soil structure maintenance by binding soil particles [6]. The beneficial effects of glomalin extend to the soil, increasing organic C content [81], supporting the water–plant relationship [82], and reducing high levels of heavy metals in the soil [83]. Mineralization of organic matter releases mineral elements that are essential for plant nutrition, including major elements (N, P, and K) and trace elements (i.e., Zn, Fe, and Cu) [84]. Our results indicate that the mineral composition of the soil was positively affected by the application of BioS. Available P, K, and Mg significantly increased, particularly when the soil was inoculated with PGPR (R) and/or AMF (A) and/or compost (C), matching their controls. These findings are consistent with the study conducted by Desoky et al. [85] and can be attributed to the increase in the field capacity and cation exchange capacity (CEC) of the soil facilitated by humic substances [86].

Altogether, the general pattern observed in the data set is that soil amendment is reflected in better growth and better soil properties, and is also reflected in salt stress-related properties of photosynthesis, gas exchange, or compound accumulation. It is noteworthy that the combined application of compost and *Bacillus* species emerges as the most effective biostimulant for enhancing sweet corn tolerance to salinity stress. This biostimulant combination significantly improves plant growth, including substantial increases in aerial and root dry weights under severe salt stress conditions. The synergistic effect of C and R reduces oxidative stress, as evidenced by reduced H_2_O_2_ levels and increased POX activity. Furthermore, this combination enhances soil quality by promoting better soil structure and mineral composition, with notable increases in soil organic C and available P. The presence of glomalin, an indicator of soil health, significantly rises under severe salt stress when using the C and R combination. The success of this biostimulant combination can be attributed to the complementary actions of compost in improving soil structure and nutrient availability, along with the beneficial impact of *Bacillus* species on plant growth and stress responses.

## 4. Materials and Methods

### 4.1. Biostimulants Preparation

Two indigenous bacterial strains, *Bacillus subtilis* and *Bacillus* sp., isolated from the rhizosphere of the arid zone of the Tafilalet palm grove in Morocco, were used. Each strain was purified and grown in tryptic soy broth (TSB) liquid medium with agitation for 48 h at 30 °C to a concentration of 1 × 109 CFU/mL. Sweet corn plants were inoculated with 10 mL (most effective volume in achieving successful root system infection) of the bacterial suspension in the root zone to ensure root system infection. The same volume was reapplied after two weeks. The selected strains were tested for tolerance to NaCl, and then in vitro plant growth-promoting characteristics were evaluated.

From the same region, the Tafilalet palm grove, a native AMF consortium containing 15 species (*Acaulospora delicata*, *Acaulospora leavis*, *Acaulospora* sp., *Claroideoglomus claroideum*, *Glomus aggregatum*, *Glomus clarum*, *Glomus claroides*, *Glomus deserticola*, *Glomus heterosporum*, *Glomus macrocarpum*, *Glomus microcarpum*, *Glomus versiforme*, *Glomus* sp, *Rhizophagus intraradices*, and *Pacispora boliviana*) was used. Inoculation was performed by adding 15 g of the inoculum (hyphae, vesicles, roots, and spore-containing substrate) (amount was found to be optimal for promoting mycorrhizal colonization and enhancing plant growth and development) during the planting of sweet corn by inserting it into the planting hole before the corn seed. Olive pomace and green waste (quack grass) collected in Marrakesh, Morocco were used as organic additives [87]. The physicochemical characteristics of the compost on a dry matter basis were as follows: total organic matter (OM) 64.0%; available phosphorus (AP): 359.1 mg kg^−1^; N: 2.1%; C/N ratio: 17.31; pH: 9.0; electrical conductivity (EC): 3.9 mS/cm; total organic carbon (TOC): 35.9%; maturity (E4/E6): 3.0. The compost was applied at a dose of 5%.

### 4.2. Plant Growth Conditions

The experiments were conducted in the greenhouse of the Faculty of Sciences Semlalia, Cadi Ayyad University, Marrakesh, Morocco, with an average temperature of 24 °C, an average relative humidity (RH) of 68.5%, and light intensity of 500 μm^−2^ s^−1^. Before transplanting, the seeds were disinfected in 10% (*w*/*v*) NaClO for 10 min and then washed with sterile distilled water. After germination in Petri dishes at a temperature of 24 °C for 4 days, the germinated seeds were transplanted into 3 kg plastic bags filled with previously sterilized soil at 180 °C (4 h). The soil had the following physico–chemical characteristics: N: 0.9 mg g^−1^, EC: 0.2 mS cm^−1^, and pH: 8.6, sand: 51%, silt: 30%, clay: 19%, AP: 11 mg kg^−1^, organic carbon: 0.6%, organic matter: 1%.

### 4.3. Treatments and Study Design

We investigated the efficacy of select microorganisms and compost on sweet corn’s tolerance to different salinity levels (control; 0 mM NaCl, moderate stress; 50 mM NaCl, and severe stress; 100 mM NaCl) and eight BioS treatments. The applied salinity levels were selected based on the sweet corn seed germination test (Figure S1). Salinity irrigation started with 25 mM NaCl to avoid the risk of osmotic shock. The BioS treatments included a control group of plants without any BioS, plants treated with an AMF consortium (A), treatment with a bacterial consortium (R), treatment with compost (C), and four combinations between BioS (AR, RC, CA, and CAR). The sweet corn plants were randomly assigned to the 24 treatments and distributed evenly throughout the greenhouse, with 10 replicates for each treatment.

### 4.4. Growth Assessment, Mycorrhization Rate, and Mycorrhizal Dependency

To determine the frequency and intensity of mycorrhization, plant roots were cleaned and stained according to the method of Phillips and Hayman [88]. Mycorrhization frequency (MF %) was calculated as (number of mycorrhizal fragments/total fragments) × 100. Mycorrhization intensity was analyzed using a range of colonization intensity scores from zero (0) to five (5) [89]. MI % = (95n5 + 70n4 + 30n3 + 5n2 + n1)/total fragments, where n5 = number of fragments scored 5 (>to 90%); n4 = number of fragments scored 4 (>to 50%); n3 = number of fragments scored 3 (<to 50%); n2 = number of fragments scored 2 (<to 10%); and n1 = number of fragments scored 1 (<to 1%). Mycorrhizal dependency (MD) was calculated by determining the ratio of the dry weight of mycorrhizal seedlings to the dry weight of non-mycorrhizal seedlings [90].

The leaf number (LN), shoot height (SH), and root length (RL) were determined at the end of harvest. The root dry weight (RDW) and shoot dry weight (SDW) were weighed after drying the samples at 80 °C until the weight remained unchanged.

### 4.5. Photosynthetic Efficiency and Gas Exchange Measurements

Chlorophyll fluorescence PSII (F_v_/F_m_) was measured using a fluorometer (OPTI-SCIENCE, OS30p, Hudson, NH, USA) [91]. Stomatal conductance (g_s_) was measured using a porometer (CI-340, Handheld Photosynthesis System, Pullman, WA, USA) following the procedure of Harley et al. [92].

### 4.6. Water Content Assessment

The water content (WC) of the sweet corn plants was determined using the following formula: $WC=\frac{FM-DM}{DM}$. The results were expressed as g H_2_O g^−1^ DM.

FM refers to the mass of fresh matter and DM refers to the mass of dry matter.

### 4.7. Antioxidant Activity Determination

To determine antioxidant enzyme activities, the method of Tejera García et al. was followed [93]. Frozen samples of the aerial part (0.1 g) were ground in a mortar with 4 mL of 1 M phosphate buffer (pH 7), 2.5% insoluble polyvinylpolypyrrolidone (PVPP), and 0.1 mM EDTA under non-denaturing conditions. The recovered supernatant was used to measure the antioxidant enzyme activities given below.

Peroxidase (POX) activity was measured as described by Polle et al. [94]. The reaction mixture contained 2 mL of phosphate buffer (pH 7, 0.1 M), 1 mL of gaiacol (20 mM), 0.3% H_2_O_2_ (10 mM), and 0.1 mL of the enzyme extract. POX activity was determined at 470 nm. Polyphenol oxidase (PPO) activity was determined using the method of Hori et al. [95]. The reaction medium contained 2 mL of catechol (10 mM) in phosphate buffer (pH 7) and 0.1 mL of enzyme extract. The PPO activity was expressed in units of enzyme mg^−1^ protein. One unit of PPO activity was defined as the amount of enzyme that caused an increase in absorbance of 0.001 min^−1^ at 420 nm.

### 4.8. Hydrogen Peroxide Content

The concentration of hydrogen peroxide (H_2_O_2_) in leaves was measured according to the method of Velikova et al. [96]. Subsamples (0.1 g) of previously frozen leaf powder were homogenized with 2 mL of 10% (*w*/*v*) trichloroacetic acid (TCA), and the mixture was centrifuged at 15,000× *g*. The supernatant (0.5 mL) was collected for determination of H_2_O_2_ content, and 0.5 mL of potassium phosphate buffer (10 mM, pH 7) and 1 mL of potassium iodide (1 M) were added. After incubation for 3 min, a standard curve of H_2_O_2_ was elaborated based on the absorbance recorded at 390 nm.

### 4.9. Soil Quality

At harvest, soil samples were collected from the root zone of the sweet corn plants and analyzed. The pH was measured with a pH meter (HI 9025, Woonsocket, Rhode Island, USA), and the electrical conductivity (EC) was measured with a conductivity meter (HI-9033, Hanna Instruments, Woonsocket, Rhode Island, USA). The available phosphorus (AP) was analyzed according to the method of Olsen and Sommers [97]. The TOC and OM were assessed according to the method described by Aubert [98], which involves the oxidation of OM with potassium dichromate in the presence of sulfuric acid. The total glomalin-related soil protein (T-GRSP) was measured according to the method of Cornejo et al. [99]. We used 4 mL of 50 mM sodium citrate buffer (pH 8.0) to extract the T-GRSP from 1 g of soil. The extract was autoclaved at 121 °C for 1 h and centrifuged at 10,000× *g* for 1 h. The T-GRSP content was evaluated according to the Bradford assay [100].

A scanning electron microscope (SEM) coupled to an X-ray spectroscopy (EDX) detector was used to evaluate the mineral element composition of the soil and its structure.

### 4.10. Statistical Analysis

The data are presented as means ± SE (standard error) and were analyzed using a multivariate analysis of variance (MANOVA) using SPSS v. 23 software (IBM, Armonk, NY, USA) to determine the interaction between the factors tested. Tukey’s honest significant difference test was applied to the data to determine the 5% significance level. Lower numbers indicate significant differences between treatments at the *p* ≤ 0.05 level. Principal component analysis (PCA) was performed with XLSTAT v. 2014 to compile all the information from the growth, physiological, biochemical, and soil physicochemical characteristics.

## 5. Conclusions

The application of BioS in sweet corn rhizospheres has demonstrated substantial benefits in improving salinity tolerance. The combination of AMF with compost significantly improved plant growth, particularly aboveground and root biomass, under high salinity conditions. Additionally, BioS positively affected gas exchange, photosynthesis, and antioxidant enzyme systems, thereby mitigating salinity-induced oxidative stress. Importantly, BioS resulted in significant soil improvements, including enhanced organic matter content, improved soil structure, and positive influences on mineral composition (i.e., K, Mg, and AP). While these results are promising, further research is required to unravel the molecular mechanisms underlying the maize stress tolerance in response to cooperative BioS interactions. Based on our findings, we recommend the combined application of compost with AMF and/or PGPR as a sustainable strategy to protect maize in agricultural settings from salinity effects and to enhance crop productivity and resilience.

## Figures and Tables

**Table 1:** Influence of different salinity levels (0, 50, and 100 mM NaCl) and biostimulant applications on growth and mycorrhization parameters in sweet corn. The treatments include control plants, sweet corn plants amended and inoculated with single biostimulants (compost (C), native arbuscular mycorrhizal fungi consortium (A), and bacterial consortium (R)), double combinations (AR: AMF + R consortia, RC: R consortium + compost, CA: compost + AMF consortium), and a triple combination (CAR: compost + AMF + R consortia). The data represent the mean ± SE of 5 biological replicates. Means with the same letter are not significantly different at *p* < 0.05 (Tukey’s HSD). SH: shoot height; RL: root length; SDW: shoot dry weight; RDW: root dry weight; MI: mycorrhizal intensity: MF; mycorrhizal frequency; MD: mycorrhizal dependency.

Treatments		Leaf Number	SH (cm)	RL (cm)	SDW (g)	RDW (g)	MI (%)	MF (%)	MD (%)
0 mM NaCl	Control	6.33 ± 0.33 bc	26.33 ± 1.85 d–f	21.33 ± 0.88 e	0.85 ± 0.08 b–e	0.16 ± 0.02 b–e	0.00 ± 0.00	0.00 ± 0.00	0.00 ± 0.00
	R	7.33 ± 0.88 a–c	37.00 ± 2.88 a–d	38.66 ± 3.48 a–d	1.05 ± 0.16 a–e	0.24 ± 0.03 a–d	0.00 ± 0.00	0.00 ± 0.00	0.00 ± 0.00
	A	6.33 ± 0.33 bc	38.66 ± 2.18 a–c	37.33 ± 1.45 a–d	1.12 ± 0.07 a–e	0.36 ± 0.00 a	1.03 ± 0.20 bc	46.66 ± 7.69 cd	23.51 ± 1.16 d
	C	7.66 ± 1.20 a–c	37.33 ± 2.90 a–d	41.50 ± 2.02 a–d	1.48 ± 0.07 a–c	0.18 ± 0.05 b–e	0.00 ± 0.00	0.00 ± 0.00	0.00 ± 0.00
	AR	7.00 ± 0.57 a–c	40.66 ± 1.45 a	35.33 ± 1.76 a–d	0.99 ± 0.20 a–e	0.28 ± 0.03 ab	1.26 ± 0.20 bc	40.00 ± 7.69 d	13.46 ± 1.16 e
	RC	8.66 ± 0.88 a–c	36.33 ± 2.60 a–e	37.66 ± 3.92 a–d	1.86 ± 0.20 a	0.25 ± 0.01 a–c	0.00 ± 0.00	0.00 ± 0.00	0.00 ± 0.00
	CA	6.66 ± 0.33 bc	39.66 ± 4.09 ab	40.33 ± 0.88 a–c	1.34 ± 0.09 a–d	0.13 ± 0.01 c–e	17.23 ± 3.60 a	86.66 ± 3.84 a	37.15 ± 3.76 c
	CAR	9.00 ± 0.57 ab	40.66 ± 2.60 a	43.00 ± 0.57 a	1.54 ± 0.09 a–c	0.16 ± 0.01 b–e	12.76 ± 3.47 a–c	82.22 ± 11.75 a	45.56 ± 1.55 c
50 mM NaCl	Control	5.33 ± 0.33 c	25.66 ± 1.45 ef	27.33 ± 3.38 d–e	0.34 ± 0.08 e	0.11 ± 0.03 de	0.00 ± 0.00	0.00 ± 0.00	0.00 ± 0.00
	R	6.33 ± 0.33 bc	31.33 ± 0.66 a–f	41.33 ± 0.88 ab	0.92 ± 0.10 b–e	0.19 ± 0.02 b–e	0.00 ± 0.00	0.00 ± 0.00	0.00 ± 0.00
	A	6.00 ± 0.57 bc	29.66 ± 2.18 a–f	35.00 ± 3.21 a–d	0.62 ± 0.10 c–e	0.09 ± 0.01 e	1.86 ± 0.93 bc	48.88 ± 4.44 bd	45.37 ± 1.69 c
	C	6.66 ± 0.33 bc	32.00 ± 2.08 a–f	28.33 ± 0.88 c–e	1.49 ± 0.27 a–c	0.11 ± 0.00 c–e	0.00 ± 0.00	0.00 ± 0.00	0.00 ± 0.00
	AR	6.00 ± 0.57 bc	29.33 ± 0.88 b–f	36.33 ± 1.45 a–d	0.65 ± 0.01 b–e	0.11 ± 0.01 de	12.93 ± 3.43 a–c	93.33 ± 3.84 a	46.61 ± 2.32 bc
	RC	10.33 ± 1.45 a	30.00 ± 1.52 a–f	37.33 ± 3.28 a–d	1.09 ± 0.11 a–e	0.13 ± 0.01 c–e	0.00 ± 0.00	0.00 ± 0.00	0.00 ± 0.00
	CA	5.66 ± 0.33 bc	27.33 ± 1.76 d–f	28.00 ± 2.08 de	1.31 ± 0.30 a–d	0.12 ± 0.00 c–e	16.05 ± 5.05 ab	75.55 ± 8.88 a–c	74.81 ± 1.21 a
	CAR	6.66 ± 0.88 bc	31.33 ± 1.45 a–f	35.33 ± 1.45 a–d	1.57 ± 0.23 ab	0.14 ± 0.00 c–e	17.06 ± 5.16 a	77.77 ± 8.01 ab	75.46 ± 1.98 a
100 mM NaCl	Control	5.33 ± 0.33 c	24.00 ± 1.15 f	28.00 ± 2.00 de	0.42 ± 0.03 de	0.07 ± 0.03 e	0.00 ± 0.00	0.00 ± 0.00	0.00 ± 0.00
	R	6.33 ± 0.33 bc	30.00 ± 1.15 a–f	35.33 ± 0.88 a–d	0.88 ± 0.11 b–e	0.14 ± 0.00 c–e	0.00 ± 0.00	0.00 ± 0.00	0.00 ± 0.00
	A	6.00 ± 0.00 bc	29.66 ± 1.85 a–f	39.33 ± 0.88 a–d	0.76 ± 0.01 b–e	0.15 ± 0.00 b–e	15.4 ± 5.83 ab	75.55 ± 8.01 a–c	44.61 ± 0.90 c
	C	6.00 ± 0.00 bc	30.33 ± 1.20 a–f	39.00 ± 0.57 a–d	1.37 ± 0.16 a–c	0.12 ± 0.03 c–e	0.00 ± 0.00	0.00 ± 0.00	0.00 ± 0.00
	AR	5.66 ± 0.66 bc	32.33 ± 0.88 a–f	38.33 ± 1.45 a–d	1.35 ± 0.41 a–c	0.15 ± 0.01 b–e	23.76 ± 4.48 a	80.00 ± 3.84 a	68.62 ± 1.74 a
	RC	7.66 ± 0.33 a–c	28.66 ± 2.60 b–f	37.33 ± 1.85 a–d	1.43 ± 0.19 a–c	0.24 ± 0.04 a–d	0.00 ± 0.00	0.00 ± 0.00	0.00 ± 0.00
	CA	6.33 ± 0.66 bc	35.66 ± 1.85 a–e	30.66 ± 1.76 b–e	1.03 ± 0.15 b–e	0.11 ± 0.02 c–e	24.15 ± 3.66 a	93.33 ± 3.84 a	56.01 ± 2.84 b
	CAR	6.33 ± 0.66 bc	28.33 ± 2.18 c–f	39.00 ± 3.46 a–d	0.68 ± 0.10 b–e	0.11 ± 0.00 de	18.2 ± 1.90 a	86.66 ± 3.84 a	38.54 ± 5.77 c

**Table 2:** Influence of different salinity levels (0, 50, and 100 mM NaCl) and biostimulant applications on the physiological parameters of sweet corn. The treatments included control plants, sweet corn plants amended and inoculated with single biostimulants (compost (C), native arbuscular mycorrhizal fungi consortium (A), and bacterial consortium (R)), double combinations (AR: AMF + R consortia, RC: R consortium + compost, CA: compost + AMF consortium), and a triple combination (CAR: compost + AMF + R consortia). The data represent the mean ± SE of 5 biological replicates. Means with the same letter are not significantly different at *p* < 0.05 (Tukey’s HSD).

Treatments		Water Content (g H_2_O g^−1^ DW)	Chlorophyll Fluorescence (F_v_/F_m_)	Stomatal Conductance (mmol m^−2^ s^−1^)
0 mM NaCl	Control	0.99 ± 0.19 fg	0.78 ± 0.00 a–d	13.73 ± 1.05 d–f
	R	4.04 ± 0.08 a–d	0.77 ± 0.01 cd	23.30 ± 5.80 a–f
	A	5.45 ± 0.36 a	0.78 ± 0.00 a–d	12.10 ± 1.44 f
	C	3.72 ± 0.61 a–d	0.79 ± 0.00 a–d	28.73 ± 2.14 a
	AR	3.80 ± 0.65 a–d	0.75 ± 0.00 d	18.62 ± 2.07 a–f
	RC	4.00 ± 0.57 a–d	0.77 ± 0.01 b–d	24.98 ± 2.15 a–e
	CA	4.60 ± 0.40 a–c	0.77 ± 0.00 cd	15.06 ± 0.35 c–f
	CAR	4.96 ± 0.57 ab	0.83 ± 0.02 ab	22.90 ± 0.40 a–f
50 mM NaCl	Control	1.24 ± 0.42 e–g	0.77 ± 0.03 a–d	11.93 ± 1.19 f
	R	3.18 ± 0.52 a–f	0.78 ± 0.00 a–d	27.43 ± 2.01 ab
	A	1.97 ± 0.26 d–g	0.77 ± 0.01 a–d	12.33 ± 1.18 f
	C	2.39 ± 0.43 c–g	0.78 ± 0.01 a–d	27.73 ± 2.14 a
	AR	3.26 ± 0.59 d–g	0.75 ± 0.00 d	13.37 ± 1.13 ef
	RC	2.35 ± 0.08 c–g	0.76 ± 0.00 cd	27.16 ± 2.43 ab
	CA	2.21 ± 0.57 d–g	0.76 ± 0.00 cd	13.83 ± 1.84 d–f
	CAR	4.05 ± 0.27 a–d	0.83 ± 0.02 a	22.33 ± 2.00 a–f
100 mM NaCl	Control	0.83 ± 0.13 g	0.77 ± 0.00 b–d	13.16 ± 1.51 ef
	R	2.39 ± 0.51 c–g	0.82 ± 0.01 a–c	24.60 ± 1.96 a–e
	A	3.46 ± 0.32 a–e	0.81 ± 0.01 a–c	13.30 ± 1.27 ef
	C	3.56 ± 0.37 a–e	0.82 ± 0.00 a–c	26.20 ± 3.59 a–c
	AR	2.75 ± 0.03 b–g	0.76 ± 0.00 cd	15.60 ± 0.63 b–f
	RC	2.75 ± 0.05 b–g	0.76 ± 0.00 cd	25.50 ± 1.70 a–d
	CA	2.19 ± 0.16 d–g	0.76 ± 0.00 cd	14.50 ± 1.89 c–f
	CAR	1.81 ± 0.70 d–g	0.77 ± 0.00 b–d	19.63 ± 3.24 a–f

**Table 3:** Influence of different salinity levels (0, 50, and 100 mM NaCl) and biostimulant applications on the soil parameters of sweet corn. The treatments included control plants, sweet corn plants amended and inoculated with single biostimulants (compost (C), native arbuscular mycorrhizal fungi consortium (A), and bacterial consortium (R)), double combinations (AR: AMF + R consortia, RC: R consortium + compost, CA: compost + AMF consortium), and a triple combination (CAR: compost + AMF + R consortia). The data represent the mean ± SE of 5 biological replicates. Means with the same letter are not significantly different at *p* < 0.05 (Tukey’s HSD). TOC: total organic carbon; TOM: total organic matter; AP: available phosphorus; EC: electrical conductivity.

Treatments		TOC (%)	TOM (%)	Glomalin (mg/g)	AP (mg/kg)	EC (mS/cm)	pH
0 mM NaCl	Control	0.67 ± 0.03 f–h	1.11 ± 0.03 hi	0.41 ± 0.02 ef	5.46 ± 0.23 jk	0.36 ± 0.03 a	7.54 ± 0.02 f
	R	0.92 ± 0.00 c–f	1.56 ± 0.00 g	0.73 ± 0.11 ef	5.34 ± 0.20 jk	0.40 ± 0.03 a	7.76 ± 0.03 b–e
	A	1.18 ± 0.08 ab	2.03 ± 0.08 a–c	0.82 ± 0.12 ef	4.54 ± 0.10 jk	0.40 ± 0.03 a	7.82 ± 0.01 a–e
	C	1.18 ± 0.00 ab	2.03 ± 0.00 a–c	0.88 ± 0.27 ef	27.44 ± 0.25 ab	0.36 ± 0.03 a	7.83 ± 0.01 a–e
	AR	1.18 ± 0.11 ab	2.20 ± 0.11 a	1.07 ± 0.17 d–f	7.82 ± 0.53 h–k	0.43 ± 0.03 a	7.83 ± 0.02 a–e
	RC	1.17 ± 0.03 a–c	2.02 ± 0.03 a–c	1.30 ± 0.15 d–f	17.54 ± 0.03 d–g	0.36 ± 0.06 a	7.80 ± 0.07 a–e
	CA	1.08 ± 0.01 a–d	1.86 ± 0.01 c–f	1.43 ± 0.23 d–f	17.97 ± 0.09 d–g	0.43 ± 0.03 a	7.68 ± 0.01 ef
	CAR	0.78 ± 0.04 e–g	1.27 ± 0.04 h	1.70 ± 0.20 c–e	12.25 ± 0.03 g–h	0.40 ± 0.05 a	7.70 ± 0.01 d–f
50 mM NaCl	Control	0.62 ± 0.03 gh	1.11 ± 0.03 hi	0.22 ± 0.13 f	6.71 ± 0.22 i–k	0.46 ± 0.03 a	7.75 ± 0.02 b–e
	R	0.98 ± 0.00 a–e	1.67 ± 0.00 e–g	0.65 ± 0.23 ef	2.48 ± 0.06 k	0.43 ± 0.03 a	7.82 ± 0.03 a–e
	A	1.07 ± 0.02 a–d	1.86 ± 0.02 c–g	1.32 ± 0.06 d–f	2.77 ± 0.18 k	0.43 ± 0.03 a	7.80 ± 0.02 a–e
	C	1.16 ± 0.02 ab	1.99 ± 0.02 a–d	1.18 ± 0.33 d–f	19.81 ± 0.26 d–e	0.46 ± 0.03 a	7.80 ± 0.05 a–e
	AR	1.00 ± 0.00 a–e	1.72 ± 0.00 d–g	1.71 ± 0.12 c–e	2.33 ± 0.21 k	0.43 ± 0.03 a	7.85 ± 0.02 a–e
	RC	1.21 ± 0.04 a	2.15 ± 0.04 ab	0.62 ± 0.19 ef	21.08 ± 0.04 cd	0.46 ± 0.03 a	7.82 ± 0.06 a–e
	CA	1.21 ± 0.01 a	2.09 ± 0.01 a–c	1.62 ± 0.12 c–e	10.32 ± 0.31 h–j	0.53 ± 0.03 a	7.90 ± 0.01 a–c
	CAR	0.88 ± 0.03 d–f	1.58 ± 0.03 fg	2.37 ± 0.36 b–d	18.67 ± 1.12 d–f	0.56 ± 0.03 a	7.92 ± 0.01 ab
100 mM NaCl	Control	0.52 ± 0.00 h	0.90 ± 0.00 i	0.69 ± 0.24 ef	13.81 ± 0.00 e–h	0.56 ± 0.03 a	7.92 ± 0.03 ab
	R	0.65 ± 0.01 f–h	1.17 ± 0.01 hi	0.73 ± 0.45 ef	12.87 ± 0.01 f–i	0.46 ± 0.03 a	7.80 ± 0.05 a–e
	A	0.96 ± 0.00 b–e	1.61 ± 0.00 e–g	1.44 ± 0.34 d–f	12.64 ± 0.01 f–i	0.53 ± 0.03 a	7.94 ± 0.03 a
	C	1.20 ± 0.02 a	2.11 ± 0.02 a–c	2.31 ± 0.50 b–d	30.68 ± 3.25 a	0.46 ± 0.03 a	7.87 ± 0.01 a–d
	AR	0.99 ± 0.00 a–e	1.74 ± 0.00 e–g	1.58 ± 0.18 c–e	12.31 ± 0.04 g–i	0.36 ± 0.06 a	7.81 ± 0.02 a–e
	RC	1.09 ± 0.00 a–d	1.98 ± 0.00 b–e	3.75 ± 0.19 a	26.70 ± 3.81 a–c	0.50 ± 0.05 a	7.74 ± 0.01 c–e
	CA	1.31 ± 0.04 a	2.30 ± 0.04 e–g	2.84 ± 0.12 a–c	23.50 ± 0.07 b–d	0.56 ± 0.03 a	7.82 ± 0.01 a–e
	CAR	1.14 ± 0.03 a–c	2.02 ± 0.03 a–c	3.17 ± 0.14 ab	32.25 ± 2.02 a	0.56 ± 0.03 a	7.94 ± 0.00 a

**Table 4:** Influence of different salinity levels (0, 50, and 100 mM NaCl) and biostimulant applications on the mineral composition of sweet corn. The treatments included control plants, sweet corn plants amended and inoculated with single biostimulants (compost (C), native arbuscular mycorrhizal fungi consortium (A), and bacterial consortium (R)), double combinations (AR: AMF + R consortia, RC: R consortium + compost, CA: compost + AMF consortium), and a triple combination (CAR: compost + AMF + R consortia). The data represent the mean ± SE of 5 biological replicates. Means with the same letter are not significantly different at *p* < 0.05 (Tukey’s HSD).

Treatments		K (%)	Ca (%)	Mg (%)	Fe (%)	Na (%)
0 mM NaCl	Control	3.67 ± 0.06 ij	4.70 ± 0.05 g	3.64 ± 0.06 d	2.76 ± 0.02 ab	0.22 ± 0.03 d–g
	R	3.35 ± 0.05 lm	3.51 ± 0.02 hi	2.89 ± 0.04 gh	2.79 ± 0.04 a	0.46 ± 0.06 cd
	A	3.76 ± 0.05 h–j	3.36 ± 0.02 ij	2.44 ± 0.05 i	0.50 ± 0.23 ij	0.01 ± 0.00 g
	C	5.68 ± 0.06 d	5.21 ± 0.03 ef	3.09 ± 0.05 e–g	0.62 ± 0.02 ij	0.01 ± 0.00 g
	AR	8.39 ± 0.05 a	9.67 ± 0.02 a	3.33 ± 0.01 e	1.60 ± 0.21 d–h	0.43 ± 0.06 cd
	RC	5.93 ± 0.01 b–d	0.91 ± 0.05 m	4.36 ± 0.03 b	2.05 ± 0.04 c–f	1.35 ± 0.05 b
	CA	4.85 ± 0.02 e	4.98 ± 0.02 e–g	3.03 ± 0.03 fg	0.63 ± 0.05 ij	0.03 ± 0.01 g
	CAR	3.41 ± 0.10 k–m	5.89 ± 0.02 d	2.51 ± 0.05 i	0.37 ± 0.05 j	0.08 ± 0.04 fg
50 mM NaCl	Control	3.32 ± 0.01 m	4.94 ± 0.02 e–g	2.64 ± 0.01 hi	1.28 ± 0.04 h	1.57 ± 0.04 b
	R	3.87 ± 0.02 g–i	3.13 ± 0.01 ij	3.76 ± 0.05 cd	2.31 ± 0.05 a–c	1.43 ± 0.05 b
	A	4.17 ± 0.02 f	7.41 ± 0.02 b	2.89 ± 0.06 gh	1.13 ± 0.02 hi	0.03 ± 0.01 g
	C	6.03 ± 0.03 bc	5.31 ± 0.04 e	6.74 ± 0.05 a	1.09 ± 0.05 hi	0.27 ± 0.06 c–g
	AR	5.03 ± 0.05 e	2.70 ± 0.26 k	3.19 ± 0.02 ef	2.45 ± 0.30 a–c	0.37 ± 0.03 c–e
	RC	6.11 ± 0.07 b	2.23 ± 0.08 l	2.55 ± 0.06 i	1.42 ± 0.06 gh	0.00 ± 0.00 g
	CA	3.56 ± 0.03 j–m	3.85 ± 0.02 h	3.15 ± 0.02 e–g	2.18 ± 0.05 b–d	0.39 ± 0.05 c–e
	CAR	4.11 ± 0.04 fg	4.87 ± 0.02 fg	2.05 ± 0.08 j	1.59 ± 0.03 d–h	0.39 ± 0.05 c–e
100 mM NaCl	Control	3.35 ± 0.02 lm	4.91 ± 0.03 fg	2.95 ± 0.05 fg	1.25 ± 0.04 h	1.60 ± 0.02 b
	R	8.43 ± 0.02 a	4.64 ± 0.13 g	3.94 ± 0.02 c	2.03 ± 0.04 c–f	1.41 ± 0.11 b
	A	5.79 ± 0.04 cd	6.02 ± 0.05 d	3.33 ± 0.03 e	1.92 ± 0.05 c–g	0.32 ± 0.04 c–f
	C	5.93 ± 0.02 b–d	6.61 ± 0.05 c	3.30 ± 0.04 e	1.60 ± 0.23 d–h	0.54 ± 0.05 c
	AR	3.57 ± 0.00 j–l	3.10 ± 0.02 jk	3.20 ± 0.05 ef	2.14 ± 0.02 cd	1.91 ± 0.04 a
	RC	5.98 ± 0.02 bc	2.18 ± 0.01 l	2.51 ± 0.06 i	1.50 ± 0.00 f–h	0.12 ± 0.02 e–g
	CA	3.61 ± 0.04 jk	3.78 ± 0.04 h	3.08 ± 0.06 e–g	2.10 ± 0.09 c–e	0.35 ± 0.05 c–f
	CAR	3.96 ± 0.02 f–h	4.85 ± 0.02 fg	1.96 ± 0.06 j	1.52 ± 0.06 e–h	0.34 ± 0.04 c–f

## Data Availability

Data are contained within the article.

## Supplementary Materials

The following supporting information can be downloaded at: https://www.mdpi.com/article/10.3390/plants12213703/s1.
