# Supplementary material for: Enhancing Maize Productivity and Soil Health under Salt Stress through Physiological Adaptation and Metabolic Regulation Using Indigenous Biostimulants
Source: Plants (Basel). 2023 Oct 27;12(21):3703. doi: 10.3390/plants12213703 (PMC10648834; doi:10.3390/plants12213703)
Supplement: Supplementary file 1 [file plants-12-03703-s001.zip › Supplementary Table S1.pdf]

Table S1. Results of multivariate analysis of variance (MANOVA) test for independent variables including salt stress (SS), arbuscular mycorrhizal fungi (A), plant growth promoting rhizobacteria (R), compost (C), and the interaction between them.

	SS	R	A	C	SS*R	SS*A	SS*C	A*R	R*C	C*A	C*A*R	SS*A*R	SS*C*A	SS*R*C	SS* C*A*R
MF%	**	NS	***	***	NS	**	**	NS	NS	***	NS	NS	**	NS	NS
MI%	**	NS	***	**	NS	**	NS	NS	NS	**	NS	NS	NS	NS	NS
MD%	***	NS	***	***	NS	***	NS	NS	NS	***	NS	NS	NS	*	*
SDW	**	NS	NS	***	NS	NS	NS	NS	NS	*	NS	NS	NS	*	NS
RDW	***	*	NS	**	NS	NS	***	**	NS	***	NS	NS	**	NS	NS
LN	**	***	NS	***	NS	NS	NS	NS	NS	NS	NS	*	NS	NS	NS
SH	***	*	***	*	NS	*	NS	NS	**	NS	*	NS	NS	NS	NS
RL	NS	***	**	**	NS	*	**	**	NS	*	***	NS	NS	NS	NS
Fv/Fm	*	NS	NS	NS	NS	NS	NS	NS	NS	NS	***	NS	NS	NS	**
WC	***	NS	***	*	NS	*	NS	**	NS	**	***	*	NS	NS	NS
g_s	NS	***	***	***	NS	NS	NS	NS	NS	*	***	NS	NS	NS	NS
PPO	***	**	NS	*	NS	***	NS	*	NS	NS	*	**	NS	NS	NS
POX	**	*	NS	**	NS	NS	NS	**	***	**	NS	***	NS	**	NS
H_2O_2	***	*	NS	**	**	*	NS	NS	NS	NS	**	***	*	NS	NS
pH	***	NS	*	NS	*	NS	NS	NS	NS	**	**	*	***	*	NS
EC	***	NS	NS	NS	NS	NS	NS	NS	NS	*	NS	NS	NS	*	NS
AP	*	NS	*	***	NS	NS	NS	NS	NS	*	NS	NS	NS	**	NS
TOC	NS	NS	***	***	NS	NS	***	***	***	***	NS	NS	**	NS	NS
TOM	NS	NS	***	***	NS	NS	***	***	***	***	NS	NS	***	NS	NS
K	NS	**	NS	NS	NS	***	NS	NS	***	***	NS	***	*	*	***
Ca	**	***	NS	***	***	***	**	***	***	NS	***	***	NS	***	***
Mg	NS	NS	***	NS	**	NS	NS	NS	**	**	NS	NS	NS	***	***
Fe	***	***	***	***	*	***	***	**	***	***	***	NS	*	***	***
Na	***	***	***	***	**	NS	***	NS	NS	NS	***	***	***	**	NS
Glom	**	NS	**	***	NS	NS	NS	NS	NS	NS	NS	NS	NS	NS	NS

MF: myorrhization frequency; MI: mycorrhization intensity; MD: mycorrhization dependency; SDW: shoot dry weight; RDW: root dry weight; LN: leaf number; SH: shoot height; RL: roots length; F_v/F_m: chlorophyll fluorescence; WC: water content; g_s: stomatal conductance; PPO: polyphenol oxidase activity; POX: peroxidase activity; H_2O_2: hydrogen peroxide; pH: hydrogen potential; EC: electrical conductivity; AP: available phosphorus; TOC: total organic carbon; TOM: total organic matter; K: potassium; Ca: calcium; Mg: magnesium; Fe: iron; Na: sodium; Glom: Glomalin; SS: salt stress; NS: not significant; *: $p < 0.05$; **: $p < 0.01$ and ***: $p < 0.001$.
