# Supplementary figures and images for: Enhancing Maize Productivity and Soil Health under Salt Stress through Physiological Adaptation and Metabolic Regulation Using Indigenous Biostimulants
Source: Plants (Basel). 2023 Oct 27;12(21):3703. doi: 10.3390/plants12213703 (PMC10648834; doi:10.3390/plants12213703)

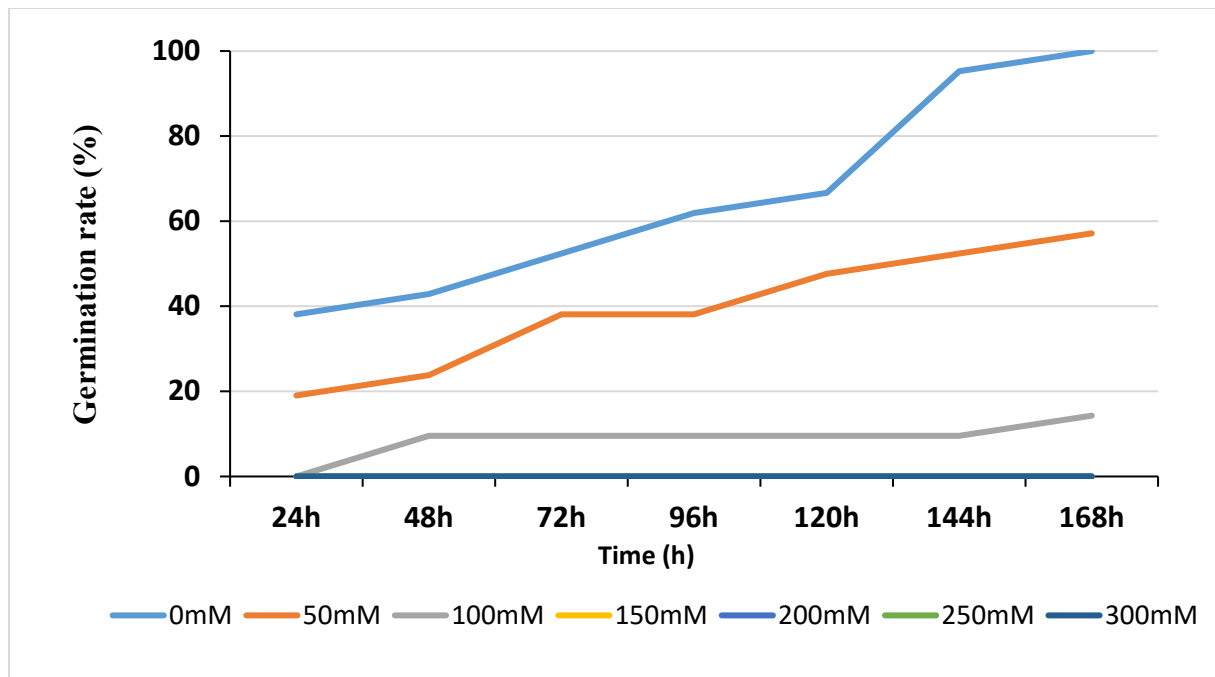

**Figure S1.** Germination rate of sweet corn seeds at varying NaCl concentrations.

Supplement: Supplementary file 1 [file plants-12-03703-s001.zip › Supplementary Figure S1.pdf]
